# Suicidal Risk, Psychopathology, and Quality of Life in a Clinical Population of Adolescents

**DOI:** 10.3389/fpsyt.2018.00017

**Published:** 2018-02-05

**Authors:** Judit Balazs, Monika Miklosi, Jozsef Halasz, Lili Olga Horváth, Dóra Szentiványi, Péter Vida

**Affiliations:** ^1^Institute of Psychology, Eötvös Loránd University, Budapest, Hungary; ^2^Vadaskert Child Psychiatry Hospital, Budapest, Hungary; ^3^Heim Pál Paediatric Hospital, Centre of Mental Health, Budapest, Hungary; ^4^Alba Regia Technical Faculty, Obuda University, Szekesfehervar, Hungary; ^5^Doctoral School of Psychology, Eötvös Loránd University, Budapest, Hungary; ^6^School of Ph.D. Studies, Semmelweis University, Budapest, Hungary

**Keywords:** suicidal risk, psychopathology, quality of life, adolescent

## Abstract

**Background:**

According to literature data, psychopathology is associated with both quality of life (QoL) and suicidal risk in adolescents, but the literature does not fully support a direct association between psychopathology and suicidal thoughts and behaviors. The aim of this study was to investigate the possible mediational role of QoL in the relationship between psychopathology and level of suicidal risk in a clinical sample of adolescents.

**Method:**

The authors examined a clinical population of 134 adolescents, aged 13–18 years. Suicidal risk—having any current suicidal ideations and/or previous suicide attempt—was assessed by the Mini International Neuropsychiatric Interview. QoL was evaluated by the adolescent self-rated versions of “Das Intervertar zur Erfassung der Lebensqualität Kindern und Jugendlichen” (ILK: Measure of Quality of Life for Children and Adolescents) and psychopathology was measured by adolescent self-rated versions of the Strengths and Difficulties Questionnaire (SDQ). A mediational model, in which QoL mediated the relationship between psychopathology and suicidal risk controlling for gender and age, was tested by means of regression analyses.

**Results:**

Gender and age were both associated with suicidal risk. Self-reported QoL significantly mediated the relationships between emotional problems (=1.846; 95% BCa CI: 0.731–2.577), as well as peer problems (=0.883; 95% BCa CI: 0.055–1.561) and suicidal risk: more emotional and peer problems were associated with lower QoL, which in turn was related to higher level of suicidal risk.

**Conclusion:**

Based on this study, which aims to make further steps in suicidal prevention, our findings suggest that clinicians should routinely screen the QoL of their patients, especially in adolescents with emotional and peer problems. Furthermore, it is important to focus intervention and treatment efforts on improving the QoL of adolescents with emotional and peer problems.

## Introduction

It is well known that about 90% of suicidal adolescents, similar to adults, have at least one psychiatric disorder [e.g., Ref. (1–4)].

During the last decade, several studies showed that children with psychiatric disorders have poorer quality of life (QoL) than their healthy peers [e.g., Ref. (5–10)]. QoL is a multidimensional construct, which aims to describe an individual’s well-being by summarizing his/her physical, mental, and social functioning (11). Though QoL includes mental state, it seeks information only about general well-being and does not screen psychopathology based on externalizing and internalizing symptoms.

On the basis of the aforementioned data, researchers aim to study the possible association between QoL and suicide, but there are conflicting available data.

There are both non-clinical and clinical studies, which support the association between QoL and suicide. A cross-sectional population study showed that adolescents above age 15 and adults with suicidal thoughts reported significantly poorer QoL than people without suicidal thoughts (12). A case–control study found that the QoL of adult suicide attempters was significantly lower than the QoL of matched controls (13). A study on a randomly selected community population found that poorer QoL was associated with higher odds of suicide ideation onset (14). A study on young adult college students also found that those with poorer QoL were more likely to endorse suicidal ideation (15). Performing a logistic regression to assess the impact of sociodemographic (e.g., age, gender, ethnicity, living satisfaction, living situation, and family SES) and clinical factors (e.g., depression) on the likelihood that young adult college students would endorse suicidality, Farabaugh et al. (15) found that poorer QoL was still a significant predictor of suicidal ideation. The results of a longitudinal epidemiological study showed that adults’ baseline self-reported life dissatisfaction was associated with a higher risk of completed suicide throughout the 20-year follow-up (16). A psychological autopsy study found that low QoL within the month before death was a significant predictor of completed suicide (17). Musyimi et al. (18) reported that based on their cross-sectional epidemiological survey conducted over a period of 3 months among adult patients seeking care from traditional and faith healers in rural Kenya, regression analysis indicated that depression, suicidal ideation, and being married predicted lower overall QoL controlling for other variables.

Investigating a clinical population, Alves Vde et al. (19) found that patients who had a mental disorder and risk of suicide attempts had lower QoL than patients without the risk of suicide attempt. Further clinical studies found that patients diagnosed with depressive disorder (20), bipolar disorder (21), or epilepsy (22) and current suicidal ideation had worse QoL than patients with the same diagnoses and without suicidal ideation. Ponizovsky et al. (23) and Xiang et al. (24) reported that the differences in the QoL of schizophrenic patients with and without previous suicide attempt remained significant after adjusting for clinical factors, e.g., age of onset of the disorder, psychiatric history, and current comorbid psychopathology variables (i.e., depressive symptoms). Moreover, both child and adult bipolar patients and adult schizophrenic patients with a history of previous suicide attempt had poorer QoL than those schizophrenic and bipolar patients who had never attempted suicide (21, 23–27). Moreover, examining epileptic patients, Andrade-Machado et al. (22) reported that in a multivariate analysis lower QoL of epileptic patients significantly increased the probability of having higher suicidal risk, next to depression. Furthermore, examining a special patient-group, depressed, or anxious family caregivers of patients with cancer, Park et al. (28) found that low QoL of family caregivers was associated with their increased odds of suicidal ideation.

However, further investigating the associations between QoL and suicidal thoughts and behaviors, there are non-supportive evidence in the literature as well. Though Kao et al. (29) found that among schizophrenia patients there is a significant association between QoL and suicidal behavior—including both previous suicide attempt and current suicidal thought—this association became non-significant while controlling for depressive symptoms, with the exception of the social domain of QoL. Moreover, the study by Yan et al. (30) reported that adult schizophrenic patients with prior suicide attempt had higher social QoL than schizophrenic patients with no history of suicide attempt. Hecimovic et al. (31) reported that low QoL of epileptic patients was not related to suicide ideation in multivariate analysis, only depression.

Most of the above described studies on the role of QoL in suicidal thoughts and behaviors focused on adults; there are only very limited data on children under 18. Moreover, only a few studies investigated the role of QoL in psychopathology and suicide risk relationship and additionally these data are conflicting. Therefore, based on the knowledge gap, the aim of our study was to examine the possible mediational role of QoL—according to the above described WHOQOL Group’s definition (1995) measuring adolescents well-being by summarizing their somatic and mental health and social functioning (i.e., school, family, peer relations, and being alone)—in the relationship between psychopathology and level of suicidal risk—having any current suicidal ideations and/or previous suicide attempt—in a clinical sample of adolescents.

## Materials and Methods

### Sample

The study population was adolescents who had been referred for psychiatric assessment at the Vadaskert Child Psychiatric Hospital and Outpatient Clinic, Budapest, Hungary. Referral was done most often by parents (based on there own opinion or suggested by teachers) or pediatricians, but sometimes by patients themselves or child protection. Both symptoms of externalizing (e.g., attention deficit hyperactivity disorder, conduct disorder, oppositional disorder) and internalizing disorders (e.g., major depressive disorder, anxiety disorders) were among the reasons for referral. Subjects were recruited from inpatient side of this Institution. Adolescents over 13 and under 18 years old were included. An exclusion criterion was mental retardation in the medical history.

The study was approved by the Ethical Committee of the Medical Research Council, Hungary (ETT-TUKEB). The parents of each adolescent and adolescents older than 14 years included in this study provided written informed consent after being informed of the nature of the study.

### Measures

Suicidal risk—having any current suicidal ideations and/or previous suicide attempt—was evaluated with the suicidal module of the *Mini International Neuropsychiatric Interview KID (MINI KID)* 2.0, Hungarian version (32–35). The MINI KID identifies current suicidal risk *via* the following questions: “In the past month did you: wish you were dead?” “Want to hurt yourself?” “Think about killing yourself?” “Think of a way to kill yourself?” “Attempt suicide?” All the questions had to be answered with “yes” or “no” by the adolescents, “yes” answers scored 1, while “no” was 0. A score of 0 was considered as no risk, scores between 1 and 5 were considered as low risk, scores between 6 and 9 were considered as medium risk, and scores ≥10 were considered as high risk. To ensure inter-rater reliability, all interviewers had participated in a training course before the study, and during the study, the interviewers were regularly supervised.

Quality of life was evaluated with the Hungarian, adolescent self-reported version of the Erfassung der Lebensqualität Kindern und Jugendlichen (ILK) (Measure of Quality of Life for Children and Adolescents) scale (36, 37). ILK assesses QoL in six different domains: school, family, peer relations, being alone, somatic health, and mental state. It uses a 5-point Likert scale, where a higher value indicates worse QoL.

Psychopathology was assessed by the Hungarian, self-reported version of the Strengths and Difficulties Questionnaire (SDQ) (38–41). SDQ is a brief screening questionnaire. SDQ consists of five scales, each with five items: (1) hyperactivity/inattention, (2) emotional symptoms, (3) conduct problems, (4) peer relationship problems scale, and (5) prosocial behavior. The first four scales constitute the Difficulties Scales. The total difficulties score is generated by summing the scores of the Difficulties scales. Each item can be answered as “not true,” “somewhat true,” and “certainly true.” Higher scores indicate higher levels of psychopathology.

### Statistical Analyses

**Figure 1:**
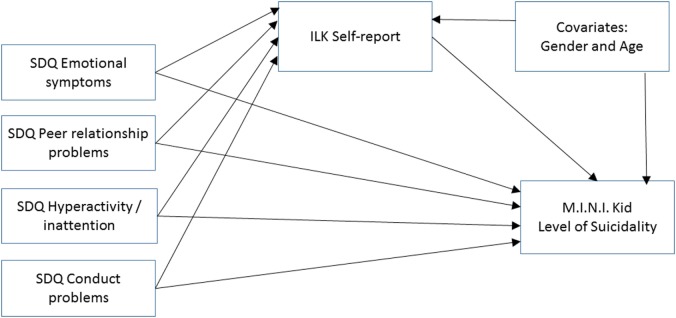
Our hypothetical mediational model: the relationship between psychopathology and level of suicidal risk (suicidal risk) as mediated by self-reported quality of life. Abbreviations: ILK, Inventar zur Erfassung der Lebensqualität von Kindern und Jugendlichen; SDQ, Strengths and Difficulties Questionnaire; MINI KID, Mini International Neuropsychiatric Interview Kid.

Descriptive statistics and intercorrelations of study variables by means of Pearson’s correlations are reported. Gender differences were explored using independent *t*-tests. According to the level of suicidal risk, we created two groups for further analyses and compared adolescents with no/low risk and medium/high risk. To test the mediational role of QoL reported on ILK in the relationships between psychopathology reported on SDQ and the level of suicidal risk measured by MINI KID, we conducted multiple logistic regression analyses using the PROCESS procedure (42). Gender and age were included as covariates throughout the analyses. Our hypothetical model is presented in Figure 1.

## Results

### Participants

A clinical sample of 134 adolescents, 72 (53.7%) boys and 62 (46.3%) girls participated in the study. Mean age was 14.48 years (SD = 1.34, range 13–18 years).

According to the MINI KID, 54 (40.3%) adolescents showed no suicidal risk, 24 (17.9%) showed low risk, 14 (10.4%) showed medium risk, and 42 (31.3%) adolescents high suicidal risk.

### Descriptive Statistics, Intercorrelations, and Reliabilities of Study Variables

Intercorrelations and reliabilities of study variables are shown in Table 1. Except for the SDQ Conduct problems scale, all measures showed acceptable to good internal consistencies.

**Table 1:** Reliabilities and bivariate relationships of study variables.

	α	skew	2.	3.	4.	5.	6.	7.
1. Child’s age	–	–	0.11	−0.02	−0.20	−22	0.10	0.19
2. SDQ emotional problems	0.78	0.07	–	0.33*	0.29*	0.24*	0.76*	0.65*
3. SDQ peer problems	0.67	0.55		–	0.02	0.20	0.61*	0.40*
4. SDQ attention deficit/hyperactivity problems	0.61	0.20			–	0.40*	0.63*	0.21
5. SDQ conduct problems	0.49	0.39				–	0.63*	0.29*
6. SDQ total difficulties	0.77	0.04					–	0.61*
7. ILK self-report	0.76	0.10						–

*N = 134*.

ILK, Inventar zur Erfassung der Lebensqualität von Kindern und Jugendlichen; SDQ, Strengths and Difficulties Questionnaire. *p < 0.008 (= 0.05/6 using Bonferroni corrections).

Inventar zur Erfassung der Lebensqualität von Kindern und Jugendlichen scores showed significant positive correlations of large effect size with SDQ Emotional problems and Total difficulties subscale scores and significant positive correlations of medium effect size with SDQ peer problems and Conduct problems scores (Table 1).

Descriptive statistics of the study variables for the total sample, and for the no/low and medium/high suicidal risk subgroups are presented in Table 2. Adolescents showing medium/high level of suicidal risk were older showed higher scores on the SDQ emotional problem scale, SDQ peer problems scale, SDQ total difficulties scale, and ILK self-report than adolescents with no/low level of suicidal risk, but no differences were found in SDQ conduct problems and SDQ ADHD subscales (Table 2).

**Table 2:** Descriptive statistics of study variables for the total sample and for no/low and medium/high suicidal risk and gender subgroups.

	Mean (SD)						
	Total sample (*N* = 134)	No/low suicidal risk (*N* = 78)	Medium/high suicidal risk (*N* = 56)	*t*(*p*) *df* = 132	Girls (*N* = 62)	Boys (*N* = 72)	*t*(*p*) *df* = 132
1. Child’s age	14.48 (1.34)	14.01 (1.14)	15.13 (1.33)	5.190 (<0.001)	14.69 (1.32)	14.30 (1.34)	1.681 (0.095)
2. SDQ emotional problems	4.60 (2.79)	3.53 (2.59)	6.10 (2.59)	5.822 (<0.001)	5.71 (2.78)	3.65 (2.44)	4.554 (<0.001)
3. SDQ peer problems	3.55 (2.42)	3.00 (2.11)	4.32 (2.62)	3.225 (0.002)	3.68 (2.61)	3.44 (2.26)	1.093 (0.580)
4. SDQ attention deficit/hyperactivity problems	4.74 (2.28)	4.47 (2.07)	4.75 (2.55)	0.016 (0.987)	4.81 (2.53)	4.69 (2.05)	0.283 (0.778)
5. SDQ conduct problems	3.27 (1.82)	3.33 (1.82)	3.17 (1.84)	0.483 (0.630)	3.39 (1.98)	3.17 (1.69)	0.696 (0.487)
6. SDQ total difficulties	16.17 (6.18)	14.62 (5.71)	18.34 (6.20)	3.593 (<0.001)	17.58 (6.65)	14.96 (5.50)	2.499 (0.014)
7. ILK self-report	22.44 (5.95)	19.22 (4.63)	26.95 (4.49)	9.650 (<0.001)	24.34 (6.20)	20.82 (5.24)	3.561 (0.001)

*ILK, Invertar zur Erfassung dcr Lchcnsqualitat von Kindcrn und Jugcnd lichen; SDQ, Strengths and Difficulties Questionnaire*.

Girls reported more emotional problems on SDQ and scored higher on ILK, indicating lower QoL (Table 2). Gender differences were found in suicidal risk as well, more girls (*N* = 46; 74.2%) than boys (*N* = 10; 13.9%) showed medium/high level of suicidal risk [χ^2^(1) = 49.802 *p* < 0.001].

### Results of the Mediational Analyses

Results of the multivariate analyses are shown in Tables 3 and 4. ILK Self-report scores were related to age, as well as SDQ emotional problems, peer problems, and conduct problems subscale scores (Table 3). On the other hand, the level of suicidal risk was significantly associated with gender, age, SDQ emotional problems, and conduct problems subscale scores, as well as ILK self-report scores (Table 4).

**Table 3:** Results of the multiple regression analyses with ILK Self-report scale as dependent.

	a	SE	*t*	*p*
Intercept	3.124	4.672	0.505	0.505
Gender (0 = girls, 1 = boys)	−0.843	0.810	−1.041	0.300
Age	0.771	0.296	2.609	0.010
SDQ emotional problems	1.040	0.163	6.401	<0.001
SDQ peer problems	0.498	0.167	2.982	0.003
SDQ attention deficit/hyperactivity problems	0.087	0.189	0.464	0.644
SDQ conduct problems	0.498	0.233	2.139	0.034

Model	*R*^2^ = 0.503, *F*(6,127) = 21.413, *p* < 0.001			

*N = 134*.

*a, unstandardized regression coefficient; ILK, Inyontar zur Erfassung dcr Lchcnsqualitat von Kindcrn und Jugcnd lichen; SDQ, Strengths and Difficulties Questionnaire*.

**Table 4:** The logistic model with level of suicidal risk according to M.I.N.I. Kid (0 = no/low, 1 = medium/high) as dependent.

	b	SE	*z*	*p*
**Step 1**				
Intercept	−26.164	5.514	−4.745	<0.001
Gender (0 = girls, 1 = boys)	−4.153	0.828	5.014	<0.001
Age	1.180	0.293	4.022	<0.001
SDQ emotional symptoms	0.305	0.125	2.450	0.014
SDQ peer relationship problems	0.505	0.163	3.099	0.002
SDQ attention deficit/hyperactivity problems	−0.013	0.154	−0.082	0.935
SDQ conduct problems	−0.237	0.174	−1.363	0.173

Model	χ^2^(6) = 103.786 *p* < 0.001; Cox and Snell *R*^2^ = 0.539; Nagelkerke *R*^2^ = 0.725			

**Step 2**				
Intercept	−61.724	19.542	−3.159	0.002
Gender (0 = girls, 1 = boys)	−9.954	2.992	−3.329	0.001
Age	2.417	0.808	2.993	0.003
SDQ emotional problems	−0.847	0.372	−2.277	0.023
SDQ peer problems	0.130	0.299	0.432	0.666
SDQ attention deficit/hyperactivity problems	−0.477	0.288	−1.655	0.098
SDQ conduct problems	−1.431	0.545	−2.624	0.009
ILK self-report	1.773	0.571	3.103	0.002

Model	χ^2^(7) = 160.361 *p* < 0.001; Cox and Snell *R*^2^ = 0.693; Nagelkerke *R*^2^ = 0.932			

*N = 134*.

*b, unstandardized regression coefficient; ILK, Inventar zur Erfassung Lebensqualität von Kindern und Jugendlichen; SDQ, Strengths and Difficulties Questionnaire; M.I.N.I. Kid, Mini International Neuropsychiatric Interview Kid*.

**Figure 2:**
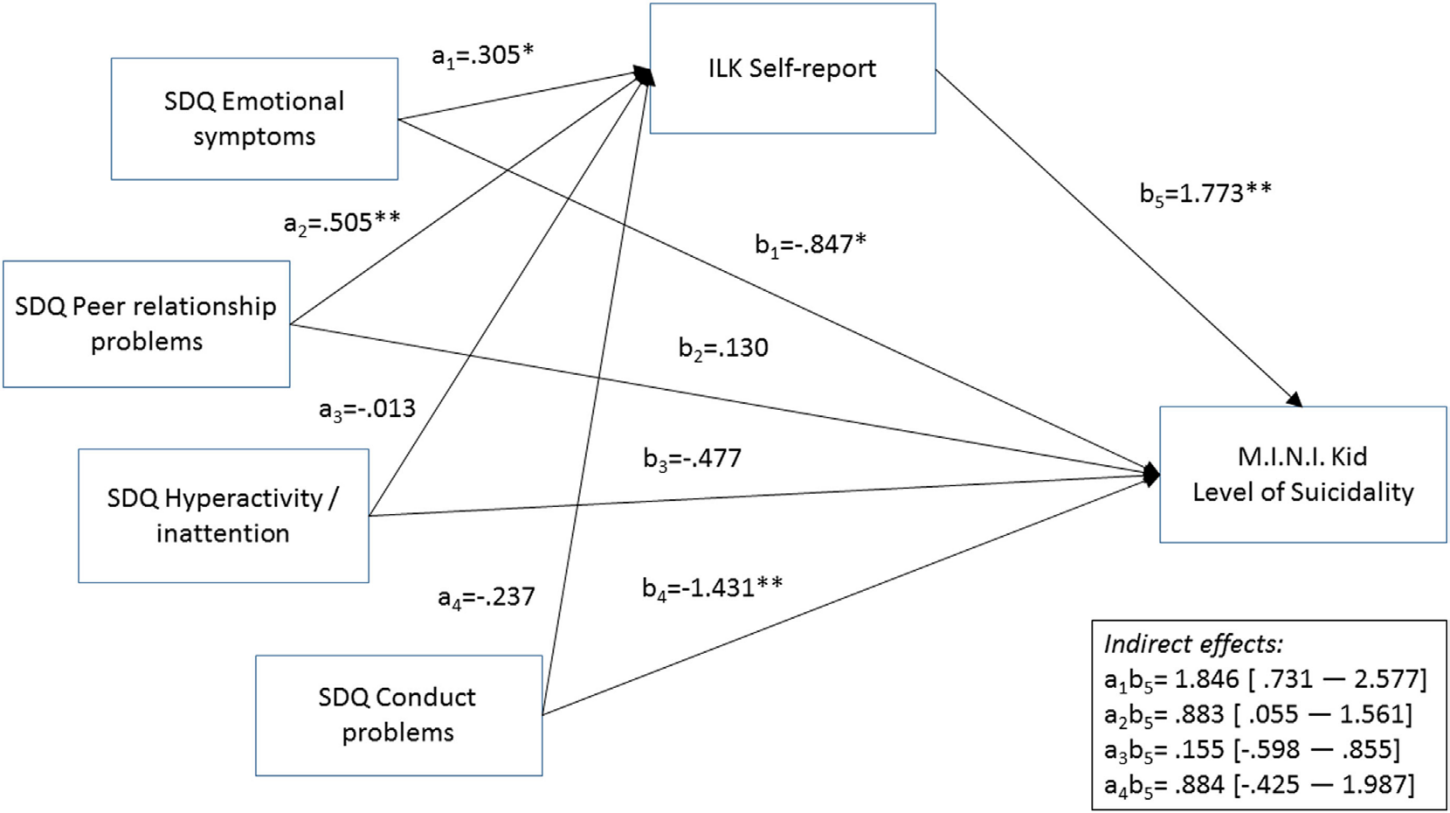
Results of the mediational analysis. *p < 0.05, **p < 0.01. *a*_j_, *b*_i_: unstandardizcd regression coefficients. Abbreviations: ILK, Inventar zur Erfassung der Lebensqualität von Kindern und Jugendlichcn; SDQ, Strengths and Difficulties Questionnaire; M.I.N.I. Kid, Mini International Neuropsychiatric Interview Kid. Gender and age were included as covariates, but are not presented in Figure because of reasons of clarity.

Inventar zur Erfassung Lebensqualität von Kindern und Jugendlichen self-report scores significantly mediated the relationships between SDQ Emotional problems (=1.846; 95% BCa CI: 0.731–2.577), as well as SDQ peer problems (=0.883; 95% BCa CI: 0.055–1.561) and suicidal risk: more emotional and peer problems were associated with lower QoL, which in turn was related to higher level of suicidal risk. The relationship between SDQ ADHD subscale (=0.155; 95% BCa CI: −0.598 to 0.855), as well as SDQ Conduct problems (=0.884; 95% BCa CI: −0.425 to 1.987) and suicidal risk was not mediated by ILK self-report (Figure 2).

## Discussion

Due to the fact that the QoL measure gains more information about daily functioning and impairment than simple symptom scoring—while it also includes non-health-related domains of functioning—there has recently been growing interest in the QoL of children with psychiatric disorders [e.g., Ref. (6, 43–45)]. However, to the best of our knowledge, this study is the first to explore QoL as a possible mediator in the relationship between externalizing/internalizing psychopathology and level of suicidal risk in a clinical sample of adolescents.

Our results are consistent with those previous studies that stated that there are gender differences already at adolescent age in several aspects: girls have more internalizing (emotional problems) psychopathology (46, 47), lower QoL (48, 49), and higher level of suicidal risk (47, 50) than adolescents’ boys.

The findings of this study are in line with previous studies as well, indicating that higher level of suicidal risk is significantly associated with both internalizing (emotional problems) and externalizing (conduct problems) psychopathology (1–4), with peer relation problems, with total difficulties (i.e., emotional + conduct + hyperactivity + peer problems) and with poorer QoL.

The very new result of this study is that QoL significantly mediates the relationships between internalizing psychopathology (i.e., emotional problems) as well as peer problems and suicidal risk: more internalizing and peer problems were associated with lower QoL, which in turn was related to higher suicidal risk.

Our data support that psychopathology and QoL are related but conceptually different constructs, showing Spearman’s correlations of small to medium effect sizes between SDQ subscales and single domains of QoL, e.g., SDQ peer problems and QoL in the peer (rho = 0.48) and being alone domains (rho = 0.01); SDQ emotional problems and QoL somatic health (rho = 0.32) and mental state (rho = 0.47).

In this study, we measured psychopathology in a dimensional way, why we share the those researchers’ opinion who have suggested during the last decade that next to categorical approaches of diagnoses, what the classification systems—i.e., Diagnostic and Statistical Manual of Mental Disorders 5th Edition (51) and International Classification of Diseases, Tenth Edition (52)—mostly follow, dimensional approaches are important for both clinical work and research (53–56). Moreover, this study supports the results of two of our previous studies, which found that subthreshold disorders—those who do not fulfill all the criteria—increase suicidal risk in children, both in the population and clinical groups of adolescents (47, 57).

These results have implications for suicide prevention. While the importance of the recognition and appropriate treatment of both internalizing and externalizing psychopathology and peer problems are well known in suicide prevention, the role of the direct measure of QoL as a possible screening method in suicide prevention is less highlighted for clinicians. Internalizing problems of a child is often less evident to others and these children often do not receive proper professional help [e.g., Ref. (58)]. The same is often true for children with peer problems, for example, victims of bullying are often ashamed and hide that they are being bullied (59, 60), additionally bullying is an important risk factor of suicide (61, 62). Based on the results of this study, screening QoL can be a marker of suicidal risk as well. Moreover, this study suggests that an improvement of the QoL, especially in the case of those who have internalizing and/or peer problems, can have a suicide prevention role. Further studies should investigate which aspects of QoL mediate the relationship between psychopathology and suicidal risk, and interventions could focus on them.

At first sight, it could be a surprising result that QoL did not mediate the relationships between hyperactivity/impulsivity/conduct symptoms and suicidal risk. However, when we made further analyses, we found that hyperactivity/impulsivity symptoms were related to more emotional symptoms and conduct problems, additionally more emotional symptoms and/or peer relationship problems were associated with higher level of suicidal risk.

We have found the same results in this study as we did in our previous study on a different clinical group of adolescents (57): the association between ADHD symptoms and suicidal risk was fully mediated by internalizing symptomatology, however it was not mediated by conduct problems. The new result is that the association between ADHD symptoms and suicidal risk was not mediated by peer problems either. All these results highlight the importance of careful assessment of comorbid internalizing symptomatology in adolescents with ADHD.

This study should be interpreted in the context of its limitations. First, the study was cross-sectional, which made us unable to consider any causal relationship among psychopathology, QoL, and suicidal risk. Second, the study population included a clinical sample. Third, although SDQ measures a wide range of adolescent psychopathology, it does not assess all of them. Third, we used self-rated scales for the assessment of QoL and psychopathology. Finally, low internal consistency of the Conduct problem subscale of the SDQ also limits the validity of the results.

In summary, according to our results, QoL significantly mediates the relationships between internalizing psychopathology, peer problems and suicidal risk. Our data indicate that suicide prevention strategies should involve assessing QoL in clinically referred adolescents, in particular in adolescents with emotional and peer problems.

## Ethics Statement

The study was approved by the Ethical Committee of the Medical Research Council, Hungary (ETT-TUKEB). The parents of each adolescent and adolescents older than 14 years included in this study provided written informed consent after being informed of the nature of the study.

## Author Contributions

JB: designed the study, trained the study staff, formulated the study questions, interpreted the results, and wrote the manuscript. MM: statistical analyses and wrote the manuscript. JH: participated in the study designed and approved the final manuscript. LH: supervised the data collection, participated in the data collection, and approved the final manuscript. DS: supervised the data collection, participated in the data collection, supervised the data collection, participated in the data entry, and approved the final manuscript. PV: participated in the data collection, supervised the data collection, participated in the data entry, and approved the final manuscript.

## Conflict of Interest Statement

The authors declare that the research was conducted in the absence of any commercial or financial relationships that could be construed as a potential conflict of interest.
